# Draft genome sequence of *Bacillus velezensis* strain Cas367 isolated from rhizosphere soil in China

**DOI:** 10.1128/mra.01027-25

**Published:** 2025-10-20

**Authors:** Xinru Wang, Yun Liu, Heli Shi, Shijie Zhang, Zhanjie Li, Jinchang Liang, Ying Wang, Lei He, Xiaoqiang Wang

**Affiliations:** 1 Key Laboratory of Tobacco Pest Monitoring & Integrated Management, Tobacco Research Institute of Chinese Academy of Agricultural Sciences 243822 , Qingdao, China; 2 Shandong Provincial Key Laboratory of Detection Technology for Tumor Markers, College of Life Science, Linyi University 165082, Linyi, China; 3 Baoji Tobacco Company of Shaanxi Province, Baoji, Shaanxi, China; 4 Enshi Tobacco Science and Technology Center, Enshi, China; 5 Science and Technology Department, China Tobacco Corporation Henan Province Company, Zhengzhou, China; DOE Joint Genome Institute, Berkeley, California, USA

**Keywords:** draft genome, *Bacillus velezensis*, rhizosphere soil

## Abstract

A bacterial strain *Bacillus velezensis* Cas367 was isolated from the rhizosphere soils of tobacco in China. Here, we present the draft genome sequence of *B. velezensis* Cas367. The genome comprises a 3,889,832 bp, with G + C contents of 46.4%.

## ANNOUNCEMENT

*Bacillus velezensis* is a gram-positive, aerobic or facultatively anaerobic bacterium within the genus of *Bacillus*, renowned for its capacity to synthesize a diverse array of antimicrobial compounds (1, 2). As a plant growth-promoting rhizobacterium, it facilitates plant development and is extensively utilized in agricultural applications as both a biopesticide and a biofertilizer (3). To advance the understanding of its genomic attributes, we isolated and sequenced the genome of a *B. velezensis* strain named Cas367. A sample was collected from the rhizosphere soil of a tobacco field in Xuan’en County (29°59″1.932″N, 109°35″2.976″E), China, on 15 June 2023. Soil suspensions were serially diluted and plated on nutrient agar (NA), followed by incubation at 28°C for 48 h. Distinct single colonies were isolated and purified through three consecutive streaks on fresh NA medium. Species identification was conducted by PCR amplification and Sanger sequencing of the 16S rRNA gene using primers 27F and 1492R (2). The obtained sequence (accession number PX410333) was analyzed using the EzBioCloud (https://www.ezbiocloud.net/). Based on 16S rRNA gene sequence analysis, isolate Cas367 exhibited 99.71% similarity to *B. velezensis* CR-502 (AY603658) and was therefore identified as *B. velezensis*.

A single colony was selected from a NA plate and inoculated into 100 mL of nutrient broth medium, followed by incubation for 24 h at 28°C with shaking at 200 rpm. Genomic DNA of strain Cas367 was extracted using a phenol/chloroform method (4). DNA purity and concentration were assessed by agarose gel electrophoresis and Qubit quantification, respectively. Sequencing libraries were constructed with the NEBNext Ultra DNA Library Prep Kit (New England Biolabs, USA) and sequenced on an Illumina NovaSeq X Plus platform using a library with an insert size of 270 bp (Illumina, San Diego, CA). The platform generated 1.64 Gb paired-end reads of 150 bp each, resulting in a 181× coverage depth. Illumina reads with a quality score <15 accounting for more than 40% of the total number of reads were filtered by fastp (5). Reads were assembled *de novo* using SPAdes v3.7 (6), and contigs <1000 bp were discarded. Default parameters for all software applications were used unless otherwise specified.

The assembled genome of strain Cas367 comprises 11 contigs with a total length of 3,889,832 bp, with an N50 of 2,020,184, a G + C content of 46.4% (Table 1). Genome annotation was performed using NCBI Prokaryotic Genome Annotation Pipeline (7), which identified 3,689 protein-coding genes. In addition, tRNA genes were predicted with tRNAscan-SE v1.3.1 (8) and rRNA operons were identified using RNAmmer v1.2 (9). The genome was found to contain 56 tRNAs and 7 rRNAs.

**TABLE 1:** Summary of genome information

Parameters	Value
Number of contigs	12
Coverage (×)	181
Total number of reads	4,705,771
Genome sizes	3,889,832 bp
G + C content	46.4%
Number of coding sequences	3,788
Number of protein-coding genes	3,689
Number of tRNA genes	56
Number of 5S rRNA genes	4
Number of 16S rRNA genes	2
Number of 23S rRNA genes	1
Genome coverage	99.26%
Completeness	99.79%
Contamination	0.00%

## Data Availability

This Whole Genome Shotgun project has been deposited at GenBank under accession JBQWFJ000000000. The version described in this paper is version JBQWFJ000000000.1. The raw sequencing reads generated from Illumina platforms have been submitted to SRA and the corresponding accession numbers are SRR35283450.
